# Twisted Integration of Complex Oxide Magnetoelectric Heterostructures via Water-Etching and Transfer Process

**DOI:** 10.1007/s40820-023-01233-z

**Published:** 2023-11-17

**Authors:** Guannan Yang, Guohua Dong, Butong Zhang, Xu Xu, Yanan Zhao, Zhongqiang Hu, Ming Liu

**Affiliations:** State Key Laboratory for Manufacturing Systems Engineering, Electronic Materials Research Laboratory, Key Laboratory of the Ministry of Education, Engineering Research Center of Spin Quantum Sensor Chips, Universities of Shaanxi Province, School of Electronic Science and Engineering, Xi’an Jiaotong University, Xi’an, 710049 People’s Republic of China

**Keywords:** Magnetoelectric heterostructures, Twist angle, Epitaxial lift-off, Magnetic anisotropy, Ferromagnetic resonance

## Abstract

- The (001)-oriented ferromagnetic La_0.67_Sr_0.33_MnO_3_ films are stuck onto the (011)-oriented ferroelectric single-crystal 0.7Pb(Mg_1/3_Nb_2/3_)O_3_–0.3PbTiO_3_ substrate with 0° and 45° twist angle.
- By applying a 7.2 kV cm^−1^ electric field, the coexistence of uniaxial and fourfold in-plane magnetic anisotropy is observed in 45° Sample, while a typical uniaxial anisotropy is found in 0° Sample.

**Abstract:**

Manipulating strain mode and degree that can be applied to epitaxial complex oxide thin films have been a cornerstone of strain engineering. In recent years, lift-off and transfer technology of the epitaxial oxide thin films have been developed that enabled the integration of heterostructures without the limitation of material types and crystal orientations. Moreover, twisted integration would provide a more interesting strategy in artificial magnetoelectric heterostructures. A specific twist angle between the ferroelectric and ferromagnetic oxide layers corresponds to the distinct strain regulation modes in the magnetoelectric coupling process, which could provide some insight in to the physical phenomena. In this work, the La_0.67_Sr_0.33_MnO_3_ (001)/0.7Pb(Mg_1/3_Nb_2/3_)O_3_–0.3PbTiO_3_ (011) (LSMO/PMN-PT) heterostructures with 45º and 0º twist angles were assembled via water-etching and transfer process. The transferred LSMO films exhibit a fourfold magnetic anisotropy with easy axis along LSMO < 110 >. A coexistence of uniaxial and fourfold magnetic anisotropy with LSMO [110] easy axis is observed for the 45° Sample by applying a 7.2 kV cm^−1^ electrical field, significantly different from a uniaxial anisotropy with LSMO [100] easy axis for the 0° Sample. The fitting of the ferromagnetic resonance field reveals that the strain coupling generated by the 45° twist angle causes different lattice distortion of LSMO, thereby enhancing both the fourfold and uniaxial anisotropy. This work confirms the twisting degrees of freedom for magnetoelectric coupling and opens opportunities for fabricating artificial magnetoelectric heterostructures.
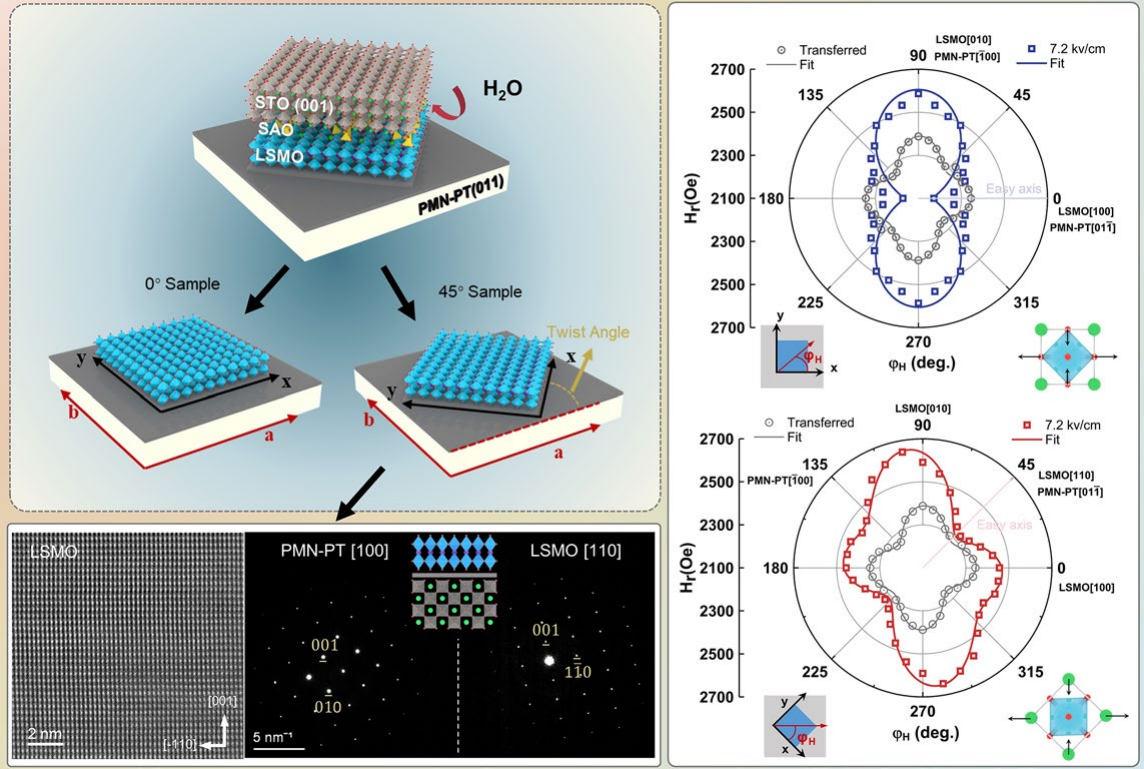

**Supplementary Information:**

The online version contains supplementary material available at 10.1007/s40820-023-01233-z.

## Introduction

The ability to control strain in magnetoelectric heterostructures has been one of the key approaches to manipulate a vast range of functional properties, since the strong electronic interactions in complex oxide materials, especially the 3d orbital electrons, make its physical phenomena sensitive to lattice deformations [1, 2]. Generally, as for strain engineering, the lattice mismatch between epitaxial film and substrate modulates the phase transitions [3], enhances ferroic order [4, 5], and modifies charge conduction [6, 7].

However, the epitaxial growth has fundamental limitations and prevents the unrestricted manipulation of strain in film. Firstly, the maximum strain is determined by lattice mismatch during the epitaxial growth [8, 9]. Secondly, because epitaxy only occurs for a relatively limited set of materials, whether the type and orientations of the materials or the strain coupling modes driven by magnetic or electric fields are restricted [10–12]. Thirdly, the clamping effect constrains the strain transfer process [13]. These reasons impose a serious obstacle to enhancing magnetoelectric coupling.

In recent years, epitaxial lift-off and transfer methods have been developed, enabling the integration of heterostructures without the restriction of crystal orientation and type. These techniques, such as chemical etching La_0.7_Sr_0.3_MnO_3_ [14], SrRuO_3_ [15], MgO [16] and SrCoO_2.5_ [17], mechanical lift-off by graphene [18], as well as water-soluble Sr_3_Al_2_O_6_ [19, 20], have produced a broad range of new heterostructures, such as CoFe_2_O_4_(CFO)/0.7Pb(Mg_1/3_Nb_2/3_)O_3_–0.3PbTiO_3_(PMN-PT), CFO/Y_3_Fe_5_O_12_(YIG) [18], La_0.67_Sr_0.33_MnO_3_(LSMO)/PMN-PT [21] and SrTiO_3_(STO)/Ce_0.8_Gd_0.2_O_1.9_(CGO) [22]. Meanwhile, by applying pressure on the thin films in direct contact with substrates, the epitaxial film can achieve one-step transfer, thus resulting in higher quality [23]. Based on these, referring to Moiré heterostructures of 2D materials [24, 25], it could be anticipated that the twist angle between ferroelectric and ferromagnetic oxide film can also extend the degrees of freedom in strain control of magnetoelectric heterostructures [26]. However, there are few related research so far.

In this work, an artificial assembly method is used to combine a (001)-oriented ferromagnetic LSMO film and a (011)-oriented ferroelectric single-crystal PMN-PT substrate, which is impossible realized through directly epitaxial growth. LSMO film is epitaxially grown on (001)-oriented SrTiO_3_ (STO)-buffered Sr_3_Al_2_O_6_ (SAO) layer by pulse laser deposition (PLD). After removing SAO layer, LSMO film is transferred to the PMN-PT with the twist angle between ferroelectric substrates and ferromagnetic films, such as 0° (0° Sample) and 45° (45° Sample). Before and after transfer, the easy axis within the thin film surface points to LSMO < 110 >, while the in-plane squareness of the magnetization curve (*M*_r_/*M*_s_) significantly increases after the lift-off process. By applying a 7.2 kV cm^−1^ electric field, a coexistence of uniaxial and fourfold magnetic anisotropy with LSMO [110] easy axis is observed for 45° Sample, which is significantly different from the uniaxial anisotropy with LSMO [100] easy axis for 0° Sample. Moreover, the in-plane angle-dependent ferromagnetic resonance field (FMR) results exhibit similar magnetic anisotropy, indicating that the 45° rotation of LSMO film on PMN-PT gives rise to different strain modes and lattice distortions from 0° Sample. This study demonstrates the twist angle degree of freedom for performance regulation in magnetoelectric heterostructures and opens up opportunities for fabricating artificial magnetoelectric heterostructures.

## Experimental Section

### Growth and Transfer of LSMO Thin Films

PLD was used to epitaxial growth sacrificial layer SAO and ferromagnetic film LSMO in turn on a single-sided polished STO (100) single crystal. The substrates' temperature was 800 °C. The pulsed laser energy was 180 mJ and the frequency was 2 Hz. Meanwhile, the O_2_ partial pressure was 20 and 30 Pa for the deposition of SAO and LSMO, respectively. Magnetron sputtering technology was used to grow electrodes of 50 nm metal Pt on the upper and lower surfaces of (011)-oriented PMN-PT single-crystal substrates. A resin glue (G1, Gatan, hardener/resin = 1:10) was used to stick as-grown LSMO films onto the platinized PMN-PT substrates at the twist angle of 45° ± 2° (45° Sample) and 0° ± 2° (0° Sample), respectively. During the transfer process, a continuous pressure was applied to the LSMO/PMN-PT heterostructures, and it was heated at 120 °C for 30 min to ensure complete curing. Finally, the sacrificial layer SAO was dissolved in deionized water for 12 h.

### Materials Characterizations

A high-resolution X-ray diffractogram (XRD) with θ − 2θ scanning (PANalytical X' Pert Pro MRD) was used to characterize the crystallinity of LSMO/SAO/STO(001) and LSMO(001)/PMN-PT(011). And an atomic force microscope (AFM) (Bruker Dimension Inc.) was used to characterize surface morphology of the LSMO/SAO/STO (001) heterostructure. Meanwhile, the microstructure and epitaxial nature of the film and interface was characterized by scanning transmission electron microscopy (STEM) (JEOL JEM-ARM 200F) with a probe spherical aberration corrector.

### Magnetic Measurement

The M–H hysteresis loops were measured by a vibrating sample magnetometer (Lake Shore 7404). An X-band (9.3 GHz) electron paramagnetic resonance system (JES-FA200) was used for FMR measurements. The sample was placed in a rectangular cavity working at the TE_102_ mode with a 360° rotating base. Electric field tuning FMR was explored by in situ poling the PMN-PT (011) along the out-of-plane direction with Pt as electrode.

## Results and Discussion

### Fabrication and Structure of Artificial Magnetoelectric Heterostructures

**Fig. 1:**
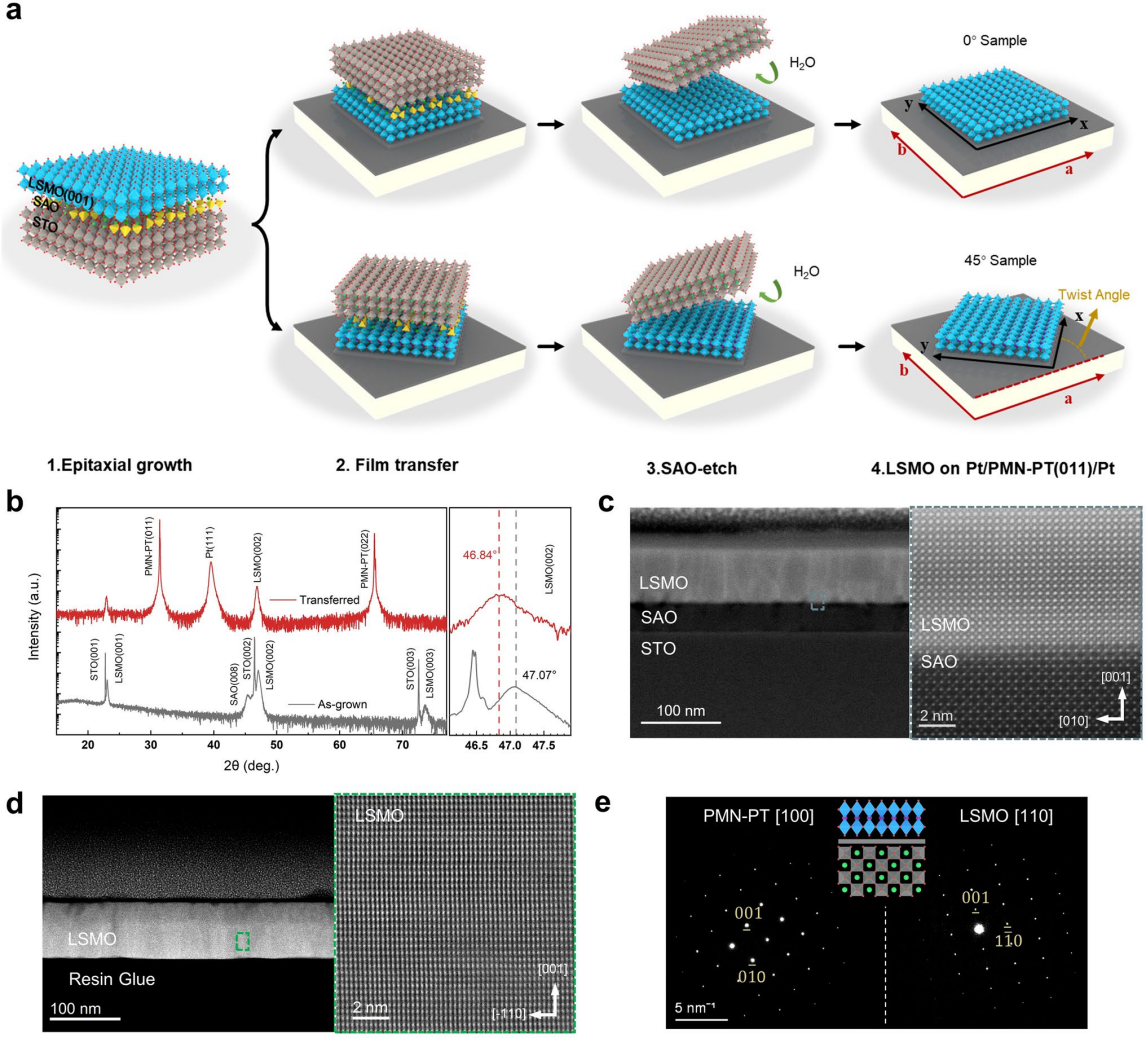
**a** Schematic diagram of LSMO film lift-off and transfer processes and structure of 0° Sample (top) and 45° Sample (down). **b** XRD patterns of the as-grown LSMO/SAO/STO (gray) and transferred LSMO on Pt/PMN-PT (red). **c** Low-magnification cross-sectional STEM image of an as-grown LSMO/SAO/STO heterostructure and high-resolution STEM image of the LSMO/SAO interface. **d** Cross-sectional TEM and high-resolution STEM images of artificial LSMO/PMN-PT heterostructures with 45° twist angle. **e** Cross-sectional selected area electron diffraction images of artificial LSMO/PMN-PT heterostructures with 45° twist angle and the schematic diagram of the cross-sectional lattice structure for 45° Sample

To obtain high-quality LSMO films, SAO (35 nm) and LSMO (70 nm) are sequentially grown on STO (001) substrates using PLD. Then, LSMO/SAO/STO(001) is stuck onto the (011)-oriented platinized PMN-PT. The PMN-PT(011)/LSMO/SAO/STO(001) is immersed in dissolved water to etch the sacrificial SAO layer. Two types of LSMO/PMN-PT are fabricated as shown in Fig. 1a (x||LSMO[100], y||LSMO[010], a||PMN-PT[01-1] and b||PMN-PT[100]). The crystallinity of LSMO/SAO/STO heterostructure as-grown and LSMO/PMN-PT transferred from STO substrate is detected by XRD as shown in Fig. 1b. Only (00*l*)-oriented diffraction peaks are found by θ–2θ scans, confirming the high crystallinity of LSMO/SAO/STO heterostructures and transferred LSMO films. All the (00*l*)-oriented diffraction peaks of LSMO films shift to the left, suggesting the increase of the lattice constant c with the disappearance of epitaxial stress after the transfer process. Figure 1c shows the low-magnification cross-sectional Z-contrast STEM images of an as-grown LSMO/SAO/STO heterostructure. High-resolution STEM image of the LSMO/SAO interface reveals that a sharp interface exists between LSMO and SAO. Uniformly distributed atoms and the consistent structure indicate that the LSMO/SAO heterostructure is coherently strained and fully epitaxial. Meanwhile, the low root mean square surface roughness of LSMO/SAO/STO heterostructure is confirmed by the AFM (Fig. S1). Figure 1d shows the cross-sectional STEM image of the transferred 45° Sample, and LSMO film is tightly stuck and has a flat interface. The high-resolution image along PMN-PT [100] exhibits the LSMO [110] lattices. Meanwhile, LSMO [110] and PMN-PT [100] diffraction pattern is observed by cross-sectional selected area electron diffraction (SAED), as shown in Fig. 1e, confirming the twist of crystal orientation in the heterostructure.

### Magnetic Evolution of As-Grown and Transferred LSMO Films

**Fig. 2:**
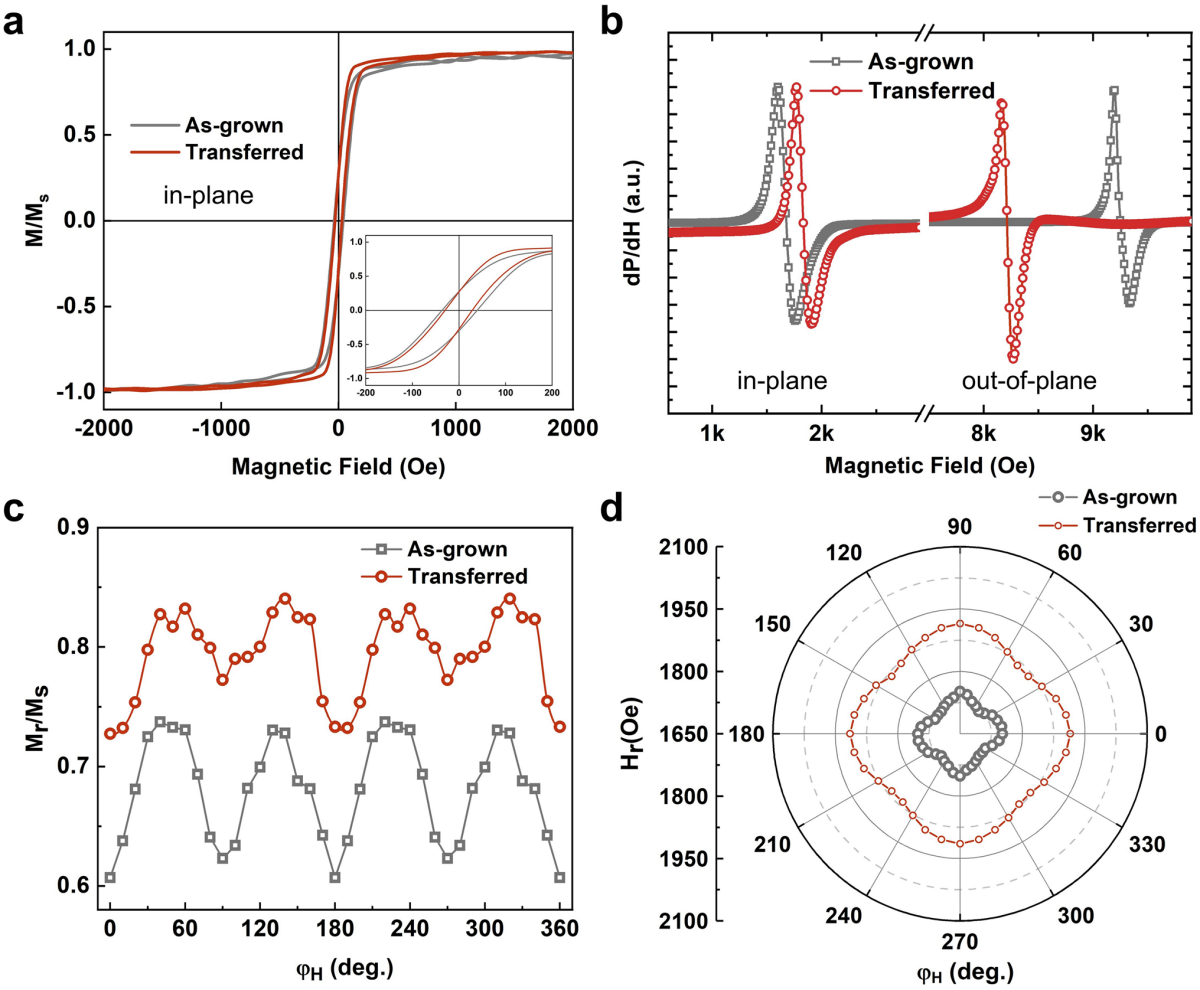
**a** Magnetization curves along the in-plane directions of as-grown (gray) and transferred (red) LSMO films, respectively. **b** In-plane and out-of-plane FMR spectra of as-grown (gray) and transferred (red) LSMO films, respectively. **c** In-plane orientation angle *φ*_H_-dependent magnetization curve squareness *M*_r_*/M*_s_. **d** In-plane orientation angle *φ*_H_-dependent *H*_r_

The in-plane magnetization curves of the LSMO films before and after the transfer process exhibit the same magnetization features with coercive field only decreasing from 39 to 25 Oe, as shown in Fig. 2a. However, for the out-of-plane, coercive field increases from 130 to 275 Oe (Fig. S2). Moreover, the obvious resonance field (*H*_r_) shift indicates the strain relaxation after the transfer, as shown in Fig. 2b. Due to the compressive stress from the STO substrate, the easy axis of the epitaxial LSMO film points to the < 110 > direction. However, the release of epitaxial strain causes 0.46% out-of-plane lattice constant increases, which could lead to a significant reduction in out-of-plane demagnetization energy (Note S1). Therefore, the *H*_r_ decreased by 1028 Oe. Besides, the biaxial in-plane anisotropy and an enhanced *M*_r_*/M*_s_ after transfer are observed, as shown in Fig. 2c. The FMR results further exhibit a slight biaxial in-plane anisotropy and a 150 Oe increase of *H*_r_ after strain release, as shown in Fig. 2d.

### Magnetoelectric Coupling of LSMO/PMN-PT

**Fig. 3:**
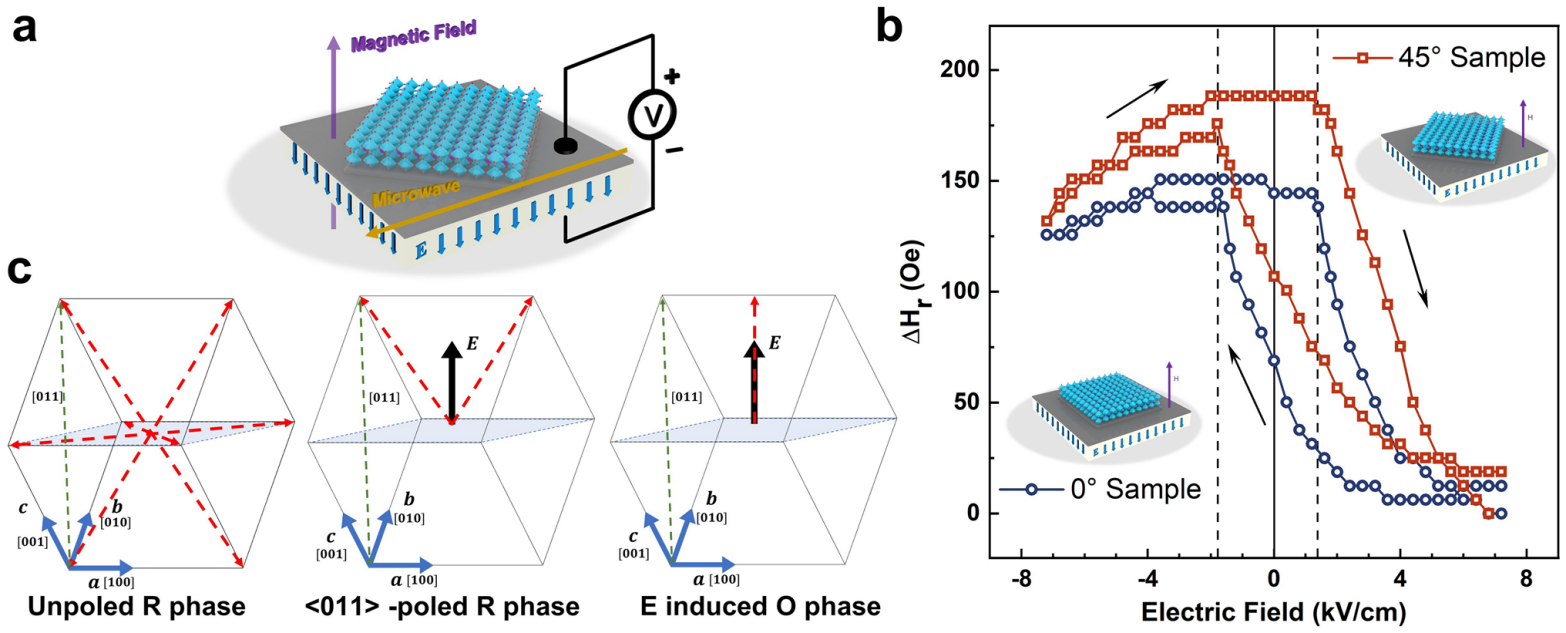
**a** Schematic diagram of FMR measurement with an applied bipolar electric field, the magnetic field points out of the plane. **b** Out-of-plane resonance field (*H*_r_) as a function of electric field (*E)* for 0° Sample (blue) and 45° Sample (red). **c** Polarizations of the PMN-PT (011) substrate upon applying an electric field along the [011] direction. The red arrows represent the polarization directions for the rhombohedral (R) and orthorhombic (O) phases

To confirm the magnetoelectric coupling of LSMO/PMN-PT, an electric field *E* is applied along the PMN-PT [011], as shown in Fig. 3a. The *H*_r_*–E* curve exhibits a loop-like behavior, as shown in Fig. 3b, which originates from the non-180° (71°and 109°) polarization switching [28] and electric-induced irreversible R–O phase transition [29]. The similar results have been reported by Liang, etc. [30, 31]. R–O phase transition induces compressive strain along the in-plane PMN-PT [100] and tensile strain along the PMN-PT [0-11]; meanwhile, polarization switching gives rise to a similar strain mode with R–O phase transition [32, 33]. Ultimately, the presence of these two mechanisms results in the loop-like curve. For both two heterostructures, the *H*_*r*_ switches at ± 1.6 kV cm^−1^ and saturates at 7.2 kV cm^−1^, which provides two nonvolatile states of high and low *H*_r_ at 0 kV cm^−1^.

The distinguishable *H*_r_ shift is synchronously observed in two different heterostructures. The maximum *H*_r_ shift along out-of-plane direction is 144.35 Oe for the 0° Sample and 188.48 Oe for the 45° Sample, corresponding to ME coefficient 20.07 and 26.18 Oe cm kV^−1^. According to the Kittel equations for out-of-plane cases [34], $f=\gamma ({H}_{r}+{H}_{k}+{H}_{eff,z}-4\pi {M}_{s})$, (3) the *H*_r_ shift can be roughly expressed as the effective field of the system's magnetoelastic energy [35], which is mainly determined by the piezoelectric coefficient on the main axis of the film and the elastic coefficient of the ferromagnetic material. These results indicate that the twist angle changes the strain applied on film, leading to the different *H*_r_ shift between two heterostructures. Moreover, an in situ XRD exhibits different peak shifts for two samples under the same electric field, which demonstrates the distinguished lattice distortion (Fig. S3). Overall, the LSMO/PMN-PT heterostructures fabricated by the epitaxial lift-off process have a loop-like *H*_r_*–E* curve. The strain applied on film could be changed by twist angle resulting in a different maximum *H*_r_ shift, which provides a possibility for tunability and nonvolatility devices.

### Electric Field Control Magnetic Anisotropy of LSMO/PMN-PT

**Fig. 4:**
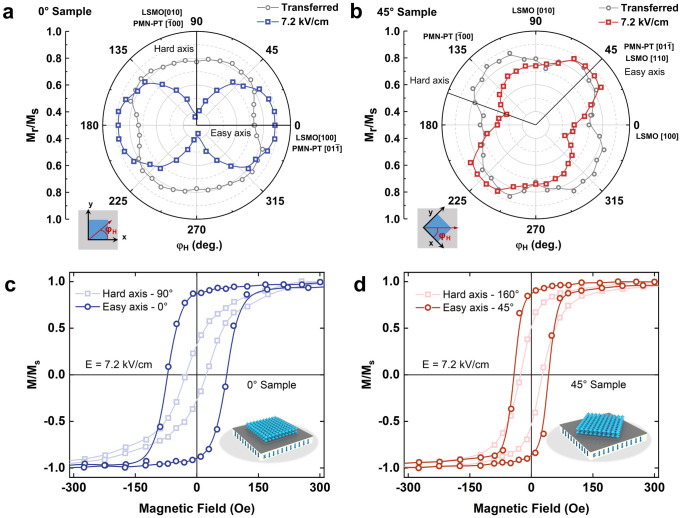
**a**, **b** Anisotropy of in-plane magnetization curves squareness (*M*_r_*/M*_s_) under 7.2 kV cm^−1^ electric field for 0° Sample and 45° Sample. The *M*_r_*/M*_s_ anisotropy of the transferred films is shown as gray line. The substrate orientation is fixed, and the structure is shown in the small schematic diagram in the lower left corner. **c**, **d** Magnetization curves of the easy axis and the hard axis for 0° Sample and 45° Sample at 7.2 kV cm^−1^. Navy blue or red is the easy axis, while light blue or red is the hard axis

To further explore the in-plane magnetic anisotropy switching, a 7.2 kV cm^−1^ electric field is applied when measuring the in-plane angle-dependent magnetization of the LSMO/PMN-PT heterostructures. In Fig. 4a, b (gray line), the *M*_r_*/M*_s_ plot after the transfer process for both 0° and 45° Samples exhibits a slight fourfold anisotropy with two magnetic easy axes LSMO [110] and LSMO [-110]. This fourfold anisotropy in transferred LSMO film originates from the incomplete release of the biaxial epitaxial tensile strain [21, 36]. When the electric field is applied, a large compression strain along the PMN-PT [100] is induced. Thus, the fourfold anisotropy of *M*_r_*/M*_s_ switches to the uniaxial anisotropy with an easy axis toward LSMO [100] for 0° Sample, as shown in Fig. 4a. However, for the 45° Sample, the twist angle between the constituent layers conduces to a different in-plane magnetic anisotropy, as shown in Fig. 4b. In this case, the easy axis appears at *φ*_H_ = 45°, coinciding with tensile strain and a positive magnetostriction coefficient of LSMO. However, the hard axis is along *φ*_H_ = 160°, which indicates a complex lattice distortion induced by different strain modes.

Figure 4c exhibits the normalized M–H loops of easy axis (φ_H_ = 0°) and hard axis (φ_H_ = 90°) for 0° Sample. The squareness *M*_r_*/M*_s_ dramatically decreases from 0.88 to 0.29 with coercivity synchronously reducing from 72.6 to 25.1 Oe when in-plane magnetic field angular (*φ*_H_) changes from 0° to 90°. Meanwhile, the coercivity field displays a similar angle-dependent feature with *M*_r_/*M*_s_ as shown in (Fig. S4). The normalized M–H loop also exhibits a different magnetization feature for 45° Sample. When the magnetic field direction changes from easy axis (φ_H_ = 45°) to hard axis (φ_H_ = 160°), the *M*_r_*/M*_s_ reduces from 0.87 to 0.53 with the coercivity field changing from 40.6 to 36.1 Oe. The differences of the magnetic anisotropy and magnetization feature at the electric field between the two samples further indicate the novel strain-induced magnetoelastic effect in the LSMO/PMN-PT heterostructures.

**Fig. 5:**
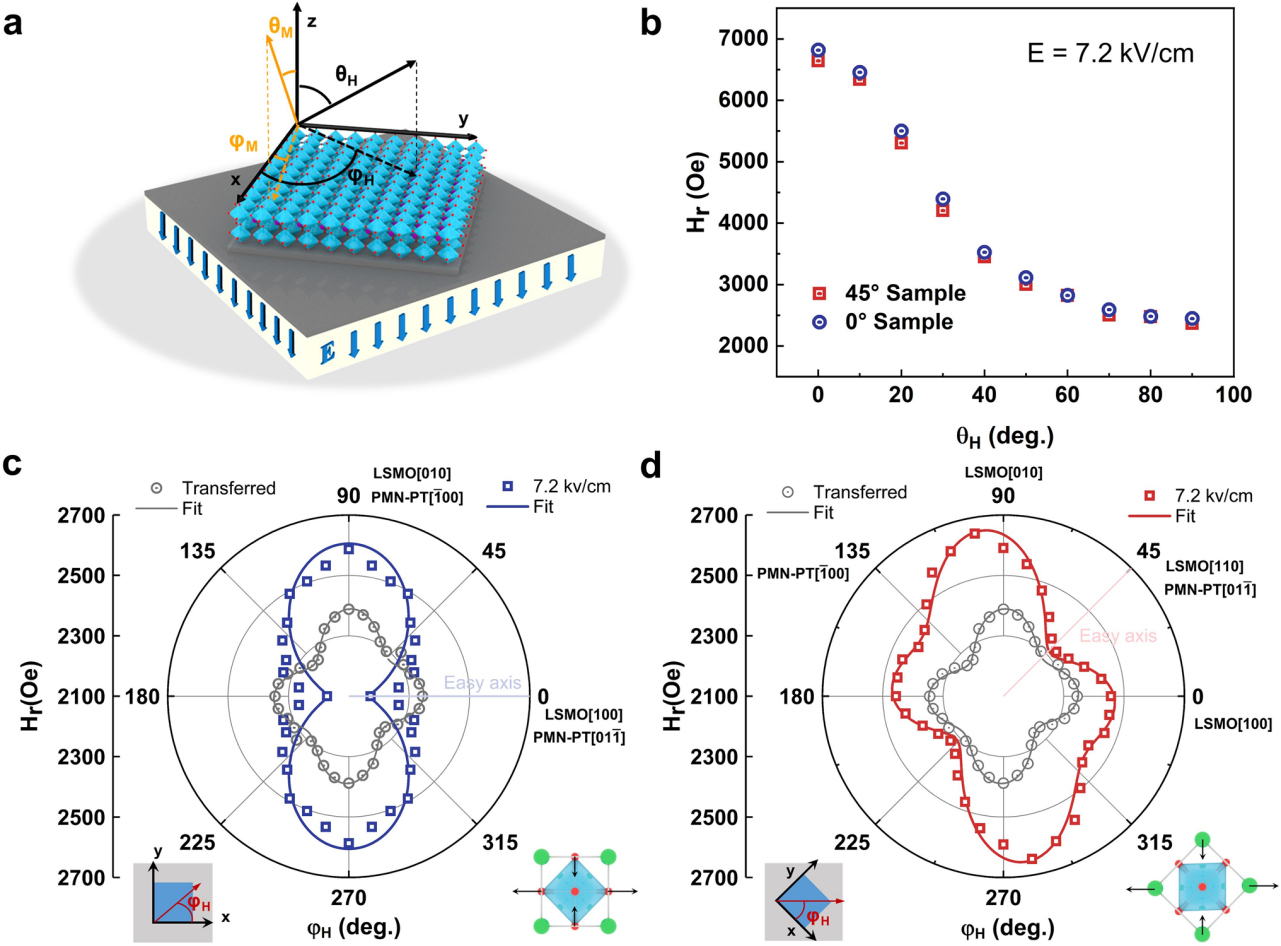
**a** Schematic diagram for polar coordinates of external magnetic field and spontaneous magnetization. The x-axis and the y-axis are parallel to the LSMO [100] and [010], respectively. **b** Out-of-plane (*θ*_H_ = 0°) angle-dependent FMR of 0° sample and 45° sample at 7.2 kV cm^−1^. **c**, **d** In-plane *H*_r_ anisotropy of the transferred and stress film at 7.2 kV cm^−1^. Points are test data; lines are fitted data. The substrate orientation is fixed, and the schematic diagram of in-plane structure and lattice distortion is shown below the plot

To further investigate the changes in magnetic anisotropy, the ferromagnetic resonance method is used. (The fitting method is shown in Note S1.) The schematic diagram of the coordinate system is shown in Fig. 5a, where the azimuth of spontaneous magnetization *M* and external magnetic field *H* with respect to the sample surface is marked as *θ*_M*,*_* φ*_M,_ and *θ*_H*,*_* φ*_H_, respectively. The out-of-plane *H*_r_ anisotropy for the two samples is not significantly different under + 7.2 kV cm^−1^, as shown in Fig. 5b, which indicates that the twist angle does not cause magnetization reorientation in out-of-plane direction. The fourfold magnetic anisotropy is observed in transferred films with easy axis along LSMO [110] and hard axis along LSMO [-110], as shown in Fig. 5c, d (gray). Fitting results demonstrate a dominant fourfold anisotropy effective *H*_*f*_, since the fitting values of *H*_*u*_ = 14 Oe and *H*_*f*_ = 33 Oe, which can be attributed to the incomplete release of epitaxial strain and the magnetocrystalline anisotropy of LSMO [27, 37].

After applying an electric field of + 7.2 kV cm^−1^, the 0° Sample changes to a uniaxial magnetic anisotropy with the easy axis along LSMO [100] and hard axis along LSMO [010], showing a dumbbell-like curve with fitting values *H*_*u*_ = 120 Oe and *H*_*f*_ = 5 Oe, as shown in Fig. 5c. However, in the 45º Sample, fitting values of *H*_*u*_ = 68 Oe and *H*_*f*_ = 55 Oe imply the coexistence of fourfold and uniaxial anisotropy in 45º Sample. The two different easy axes point to *φ*_H_ = 45° (LSMO [110]) and *φ*_H_ = 140°, while a strong hard axis is along *φ*_H_ = 100°, as shown in Fig. 5d. The term *H*_*u*_ refers to the magnetoelastic energy, which describes the uniaxial stress anisotropy due to piezoelectric strain from the substrate [38, 39]. For LSMO with a positive magnetostriction coefficient, the easy axis occurs along the tensile stress direction or perpendicular to the compressive stress direction. In 0° Sample, the compressive stress acts on LSMO [010] and tensile stress acts on LSMO [100]. Thus, as electric field applied, LSMO film displays a uniaxial magnetic anisotropy with LSMO [100] easy axis. In 45° Sample, the 45° twist angle causes the compressive stress to act on the LSMO [-110] (*φ*_H_ = 135°), while the tensile stress acts on the LSMO [110] (*φ*_H_ = 45°). Therefore, the uniaxial magnetic anisotropy rotates 45° and easy axis points to LSMO [110], due to the twist angle. Besides, this twist angle causes a *φ*_f_ = 28° phase shift between uniaxial stress anisotropy and fourfold magnetocrystalline anisotropy, which explains the new type of magnetic anisotropy in 45° Sample. This result further indicates that, by the twisted integration, the specific substrate's piezoelectric strain can produce a twist angle-dependent magnetic anisotropy in the magnetic films. This twist angle degree of freedom could help to discover new physical phenomena in artificial magnetoelectric heterostructures.

## Conclusions

In summary, we applied the epitaxial lift-off technique to stick the epitaxial (001)-oriented LSMO ferromagnetic films onto the (011)-oriented PMN-PT ferroelectric substrates with different twist angles. Due to the 45° twist angle between LSMO and PMN-PT, the biaxial strain from PMN-PT acts on the LSMO layer in different modes, resulting in the coexistence of uniaxial and fourfold in-plane magnetic anisotropy. This twist integration method provides tunability of the twist angle between different layers, allowing a new degree of twist freedom. This study demonstrates the twist angle degree of freedom for performance regulation in magnetoelectric heterostructures and confirms the great research potential of twist angle-adjustable through epitaxial lift-off technology.

## Supplementary Information

Below is the link to the electronic supplementary material.

Supplementary file1 (PDF 501 KB)
