# Supplementary material for: Twisted Integration of Complex Oxide Magnetoelectric Heterostructures via Water-Etching and Transfer Process
Source: Nanomicro Lett. 2023 Nov 17;16:19. doi: 10.1007/s40820-023-01233-z (PMC10656404; doi:10.1007/s40820-023-01233-z)
Supplement: Supplementary file 1 — Supplementary file1 (PDF 501 KB) [file 40820_2023_1233_MOESM1_ESM.pdf]

Supporting Information for

Twisted Integration of Complex Oxide Magnetoelectric Heterostructures via Water-Etching and Transfer Process

Guannan Yang[1], Guohua Dong[1,] *, Butong Zhang[1], Xu Xu[1], Yanan Zhao[1], Zhongqiang Hu[1]*, Ming Liu[1]

[1]State Key Laboratory for Manufacturing Systems Engineering, Electronic Materials Research Laboratory, Key Laboratory of the Ministry of Education, Engineering Research Center of Spin Quantum Sensor Chips, Universities of Shaanxi Province, School of Electronic Science and Engineering, Xi'an Jiaotong University, Xi'an 710049, P. R. China

*Corresponding authors. E-mail: guohuadong@xjtu.edu.cn (Guohua Dong); zhongqianghu@xjtu.edu.cn (Zhongqiang Hu)

Supplementary Figures and Table

The Atomic Force Microscope image of LSMO/SAO/STO exhibits a 1.02 nm square surface roughness. Meanwhile, the optical photo of 45º Sample exhibits the twist angle between LSMO film and PMN-PT substrate. The LSMO thin films in the 0° sample and the 45° sample were deposited simultaneously on different spatial positions. Therefore, there is no significant distinction in diffraction peaks.

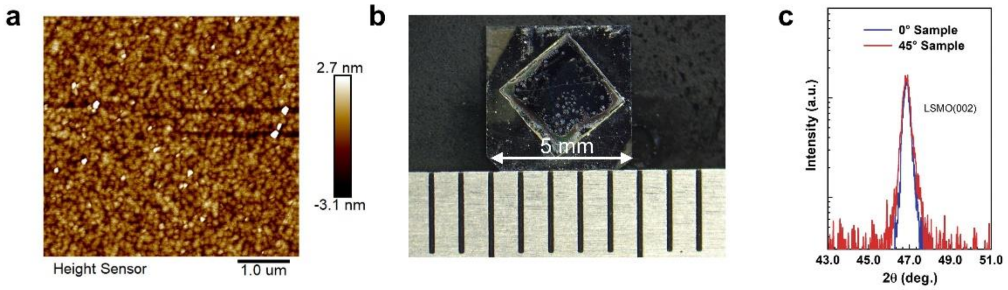

Fig. S1 (**a**) Atomic force microscope image of LSMO/SAO/STO heterostructure. (**b**) The optical photo of 45° Sample. (**c**) LSMO (002) diffraction peaks of the 45° Sample and 0° Sample after transferred

The out-of-plane magnetization curves of the LSMO films exhibit the hard-axis magnetization features after transfer. However, the coercive field increase from 130 to 275 Oe, which can be attributed to the 0.46% out-of-plane lattice constant increases after transfer.

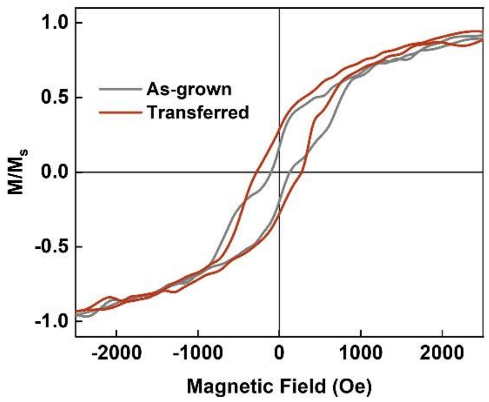

Fig. S2 The out-of-plane magnetization curves of as-grown (grey) and transferred (red) LSMO films, respectively

An in-situ XRD exhibits different peak shifts for two samples under electric field. The diffraction peak shift of 45º Sample is 0.118º with a decrease in peak intensity, while the peak shift is 0.054º with an increase in peak intensity for 0º Sample. The different shift confirms the distinguishing degree of lattice deformation, which further indicates that the twist angle changes the strain applied on film.

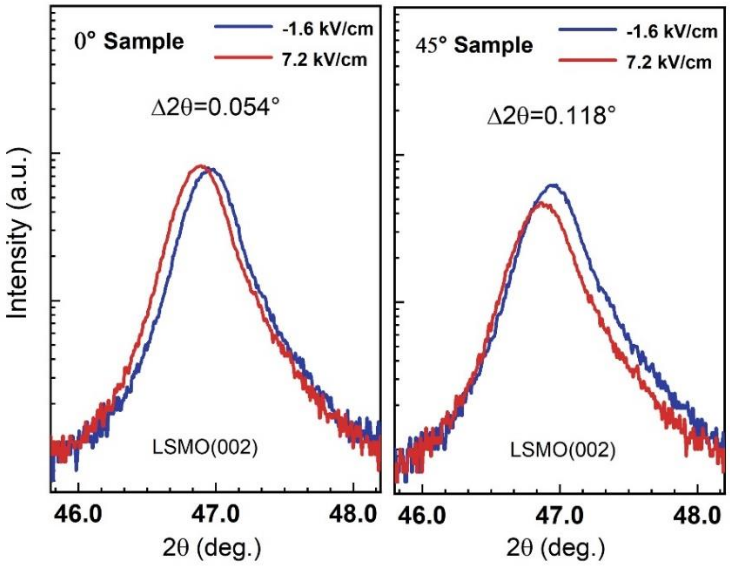

Fig. S3 LSMO (002) diffraction peak for two LSMO/PMN-PT heterostructures under -1.6 kV/cm and 7.2 kV/cm electric field

When electric field was applied, a large compression strain and a tensile strain along the PMN-PT [100] and PMN-PT [0-11] direction are induced respectively. Thus, a uniaxial coercivity field anisotropy with easy axis towards LSMO [100] for 0° Sample is observed, showing in Fig. S4a. The coercivity reduces from 72.6 to 25.1 Oe when in-plane magnetic field angular (φ_H) changes from 0° to 90°. However, for 45° Sample, the 45° twist angle changes the strain mode that applied to LSMO film, resulting in a mix coercivity field anisotropy, as shown in Fig. S4b. When magnetic field direction changes from easy axis (φ_H =45°) to hard axis (φ_H =160°), the coercivity field changes from 40.6 Oe to 36.1 Oe.

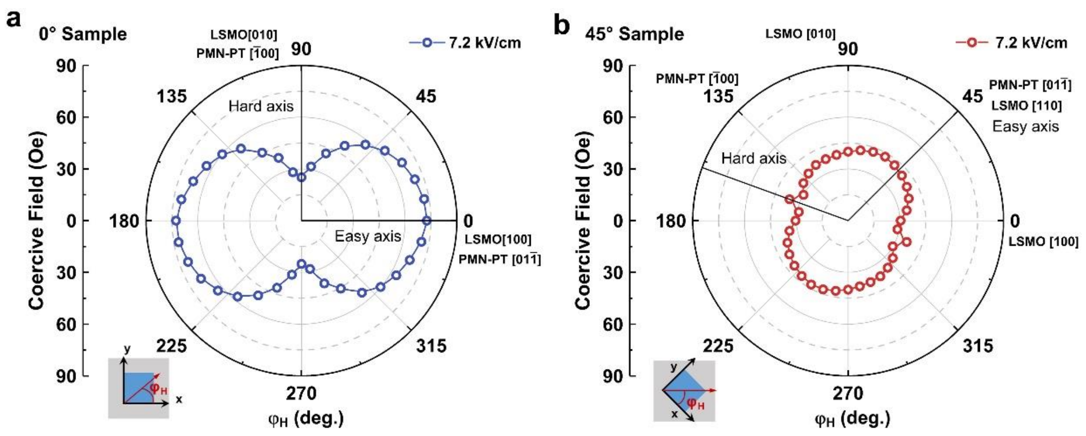

Fig. S4 The in-plane coercivity field anisotropy of the 0º Sample (**a**) and 45º Sample (**b**) at 7.2 kv/cm electric field. A similar angle-dependent feature with M_r/M_s curve is noted

Note S1 FMR Fitting

For the quantitative analysis of magnetic anisotropy energy change, the energy density of the LSMO film is given as [S1],

$$E = -M_s H[\sin\theta_M \sin\theta_H \cos(\varphi_M - \varphi_H) + \cos\theta_M \cos\theta_H] - 2\pi M_s^2 \sin^2\theta_M + K_p \sin^2\theta_M - K_u \sin^2\theta_M \cos^2(\varphi_M - \varphi_u) - \frac{K_f}{8}\left[3 + \cos 4\left(\varphi_M - \varphi_f\right)\right] \sin^4\theta_M \quad \text{(S1)}$$

where the first term is the Zeeman energy, the second term represents the demagnetization energy, the third term means the perpendicular anisotropy energy, and the last two terms are respectively in-plane uniaxial anisotropy energy and fourfold magneto-crystalline energy. In the expression, M_s is the magnetization at saturation, K_p, K_u and K_f are the out-of-plane, in-plane uniaxial and the fourfold anisotropy constant respectively, and the definition of the angle symbol is shown as Fig. 5a. Moreover, K_u and K_f are effective anisotropy constants resulting from the simultaneous occurrence of the magneto-crystalline term, the stress term, a true volume term and of the additional contribution of a surface energy term.

The resonance condition formulated by Smit and Beljers is given by the following expression [S2]:

$$\left(\frac{\omega}{\gamma}\right)^2 = \frac{1}{M_s^2 \sin^2\theta_M}\left(\frac{\partial^2 E}{\partial\theta_M^2}\frac{\partial^2 E}{\partial\theta\varphi_M^2} - \left(\frac{\partial^2 E}{\partial\theta_M \partial\varphi_M}\right)^2\right) \quad \text{(S2)}$$

where ω is the resonance frequency, $\gamma = g\mu_B/\hbar$ is the gyromagnetic ratio, g is the g factor, and μ_B is the Bohr magneton. For LSMO, the magnetic moment is entirely contributed by the electron spin, which means the g=2.0. By minimizing the total free-energy density E, it is given by

$$\frac{\partial E}{\partial\theta_M} = \frac{\partial E}{\partial\varphi_M} = 0 \quad \text{(S3)}$$

In case of an in-plane magnetic field, the magnetization M lies in LSMO film plane, and thus $\theta_M = \theta_H = 90^o$. The resonance condition can be reduced to,

$$\left(\frac{\omega}{\gamma}\right)^2 = \left[H_r \cos(\varphi_H - \varphi_M) + 4\pi Meff + \frac{1}{2} H_f\left(3 + \cos 4\left(\varphi_M - \varphi_f\right)\right) + 2H_u \cos^2(\varphi_M - \varphi_{fu})\right]\left[H_r \cos(\varphi_H - \varphi_M) + 2H_f \cos 4\left(\varphi_M - \varphi_f\right) + 2H_f \cos 2(\varphi_M - \varphi_u)\right] \quad \text{(S4)}$$

where the $4\pi M_{eff}=4\pi M_s-2K_p/M_s$ defines effective demagnetizing field, and $H_u = K_u/M_s$, $H_f = K_f/M_s$ is the in-plane uniaxial and the fourfold anisotropy effective field, respectively. Giving that the $\gamma/2\pi$ = 2.8 MHz/Oe and $f = 2\pi\omega$=9.4 GHz, the fitting results of relevant parameters are shown in the table 1.

Table S1 FMR Fitting result

	$4\pi M_{eff}$	H_u	φ_u	H_f	φ_f
Freestanding	1240±5	14±2	2/3π	33±2	0
0° Sample	1045±5	120±5	0	5±2	0
45° Sample	970±5	68±2	0	55±2	7/45π

Supplementary References

[S1] P. Ghising, B. Samantaray, Z. Hossain. Spin inhomogeneities at the interface and inverted hysteresis loop in $La_{0.7}Sr_{0.3}MnO_3/SrTiO_3$., Phys. Rev. B **101**(2), 024408 (2020). https://doi.org/10.1103/PhysRevB.101.024408

[S2] M. Belmeguenai, S. Mercone, C. Adamo, L. Mechin, C. Fur, P. Monod, P. Moch, D. G. Schlom., Temperature dependence of magnetic properties of $La_{0.7}Sr_{0.3}MnO_3/SrTiO_3$ thin films on silicon substrates. Phys. Rev. B **81**(5), 054410 (2010). https://doi.org/10.1103/PhysRevB.81.054410
